# The Relationship between Natural Park Usage and Happiness Does Not Hold in a Tropical City-State

**DOI:** 10.1371/journal.pone.0133781

**Published:** 2015-07-29

**Authors:** Le E. Saw, Felix K. S. Lim, Luis R. Carrasco

**Affiliations:** Department of Biological Sciences, National University of Singapore,14 Science Drive 4, Singapore, 117543, Republic of Singapore; Queen Mary University of London, UNITED KINGDOM

## Abstract

Previous studies have shown that contact with urban green spaces can produce positive effects on people's stress, health and well-being levels. However, much of this research has been conducted in the temperate regions of Europe or North America. Additionally, most studies have only compared the effects of urban and natural areas on health and well-being, but not made a finer distinction between different types of urban green spaces. We tested the relationship between well-being and the access or use of different types of green spaces among young adults in Singapore, a tropical city-state. The results showed that extraversion and emotional stability increased subjective well-being, positive affect and life satisfaction and decreased stress and negative affect. In addition, we found that level of physical activity increased positive affect and health problems increased negative affect. Neither access to green spaces nor the use of green spaces in Singapore significantly affected the well-being metrics considered, contradicting findings in the temperate regions of the world. We hypothesize that the differences in temperature and humidity and the higher greenery and biodiversity levels outside parks in Singapore could explain this phenomenon. Our results thus question the universality of the relationship between well-being and park usage and highlight the need for more research into the multifaceted effects of green spaces on well-being in the tropics.

## Introduction

It has been historically suggested that urban environments contribute to poor mental health and well-being [1]. Increased prevalence of psychiatric disorders, including depression, psychosis and anxiety disorders, have been reported in urban areas as compared to rural areas [2–4]. This is an issue of increasing concern as urbanization is projected to increase from 54% to 66% by 2050 [5]. Cities face a host of social problems and environmental stressors which can contribute to declines in mental health and well-being, such as overcrowding, noise and pollution [3, 6].

The high psychological demands imposed by cities have driven a desire for greater contact with nature [6]. Numerous studies have shown that contact with green spaces can produce positive effects on people's stress, health and well-being levels [7–9]. Urban residents with greater exposure to natural environments have been found to benefit from lower mental distress, reduced stress and better mood [10, 11]. Hence, as urbanization continues to spread around the world, investigations into the relationship between urban green spaces and well-being grow increasingly relevant. Understanding this can potentially help to improve urban quality of life.

**Fig 1:**
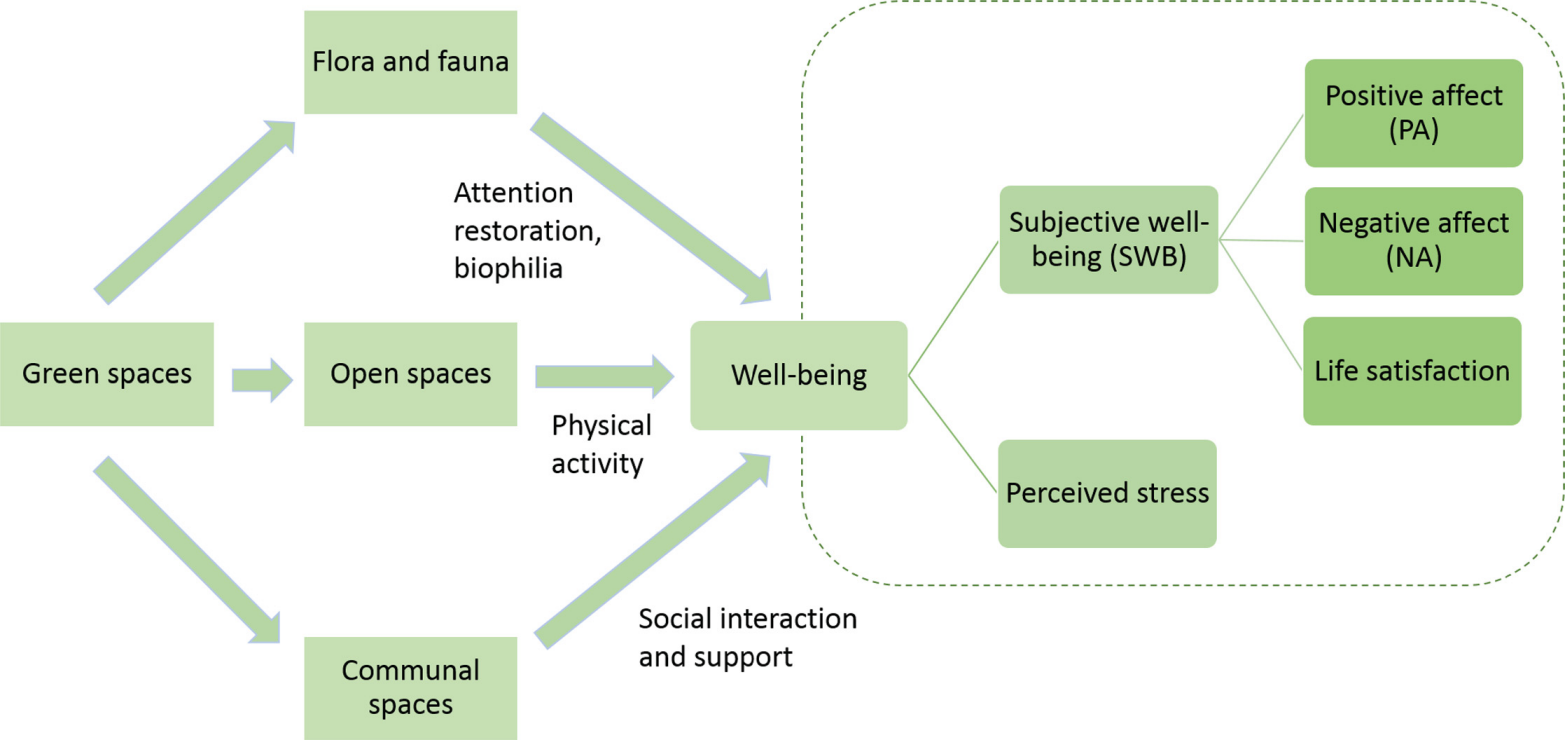
Theorised Relationships between well-being and the use of green spaces. Green spaces can affect subjective through the attention restoration and biophilia hypotheses, via physical activity and increasing social interactions and bonding. Five different aspects of well-being were then studied as a function of green space usage and accessibility.

Exposure to natural environments can lead to increased well-being via three mechanisms (Fig 1). The first is based on the Attention Restoration Theory [12], which explains that green spaces offer much potential for restoration. Cities, being multi-faceted and complex, demand much attention to deal with [13]. Over long periods of time, this can lead to stress and fatigue [14]. However, natural environments can help to restore directed attention by providing opportunities to be fascinated by the surroundings and distanced from daily routine [12]. By alleviating fatigue in attention, contact with nature can relieve irritability to improve mood and well-being [14] and potentially reduce stress [15].

The second mechanism through which green spaces may improve well-being is founded on the biophilia hypothesis [16]. This suggests that humans have an "innate tendency to focus on life and lifelike processes" [16], and that this affiliation for nature will not change even as people move to reside in urban environments [16–18]. This may be linked to Ulrich's psycho-evolutionary model of stress recovery which argued that exposure to nature triggers positive emotions as a form of attraction towards safe environments [19]. Hence, green spaces may contribute to our well-being by providing sites where biodiversity can flourish, allowing us to affiliate with nature and thereby satisfying our innate need to do so. This is supported by a finding that higher perceived species richness within urban green spaces improves visitors' psychological well-being [20]. Thus, it is likely that biodiversity and nature still remain relevant even in heavily urbanized environments. Contact with nature in green spaces satisfies our inherent biophilia, thereby improving our mood, satisfaction and well-being.

Lastly, green spaces can improve physical and mental health outcomes by promoting certain behavior that boosts well-being. Studies have shown that proximity to green spaces encourage physical activity [21, 22]. In turn, such activity within these spaces has been linked to various physical and mental health benefits, such as improved self-esteem, mood and mental health [23–25]. Additionally, provision of green spaces in the living environment also offers opportunities for contact with others. The presence of green spaces within one's neighbourhood facilitates social interaction [26] and strengthens social support [27]. This in turn is linked to improved happiness and well-being [28, 29]. Thus, through promoting physical activity and social interaction, green spaces may indirectly contribute to an improvement in well-being.

From the above, it can be established that an increase in the use and provision of urban green spaces benefits stress, happiness, health and well-being levels. However, much of the research on green spaces have been conducted in the temperate regions of the global North. The relationship between green spaces and well-being might change in the tropics due to varying climatic and environmental factors. Especially with the onset of global warming, heat and humidity are two such factors that may negatively alter the experience of using a green space. Thus, previous findings about green spaces should not be assumed to be applicable within tropical regions. There is a need to test if the relationship still holds in the tropics.

Additionally, most studies have only compared the effects of urban and natural areas on health and well-being, but have not made a finer distinction between different types of urban green spaces [30]. While studies have investigated the effect of different characteristics of urban green spaces on restoration and recovery [31, 32], these attributes have not been linked to specific categories of green spaces predetermined by urban planners. As cities become increasingly populated [33], there is a growing need to optimize land use. Thus, achieving a deeper understanding about which type of green space is best for the well-being of urban residents will be highly useful. This helps to balance the practical requirements of size and proximity to residences as urban authorities plan for park use in the city. Especially in the developing world where cities are experiencing the bulk of urban growth across the globe, this finding will be of high relevance [33].

To address these gaps in the existing literature, this study aims to test the relationship between well-being and the use of different types of green spaces within Singapore, a tropical city-state. Singapore is a small island-state 710 km^2^ in size, and it is a global city with no rural hinterland. Thus, the effects of urban greening can be deemed as particularly important since residents do not have alternatives in which to seek nature easily out of the city.

In this paper, we test three hypotheses. The first hypothesis is that an increase in the use of green spaces will improve individual well-being. Secondly, we hypothesize that living in proximity to urban green space increases well-being. Finally, different types of green spaces may produce different effects on well-being; thus, we also hypothesize that the access and use of nature reserves or regional parks will lead to improved well-being more so than that of neighbourhood parks or park connectors.

## Methods

### Data collection

#### Survey

Data for this study were collected through conducting surveys with students in the National University of Singapore. The surveys were either conducted online or in class during lecture periods. The online survey was advertised to the student population via the university intranet system and opened to all students for a period of two months. Survey questionnaires were also distributed randomly to five undergraduate lecture groups in-person. To ensure a variety of participants, two lecture groups belonged to life science modules and the remaining belonged to general university-wide modules. No personal, identifiable information was obtained from any respondent. This study was approved by the Institutional Review Board of the National University of Singapore (approval number: B-14-230E; see Survey A in S1 File for the full survey). In total, 497 students participated, out of which 426 returned complete questionnaires that were used in the final dataset. 173 responses were from the online survey and the remaining 253 responses were from surveys conducted in lectures (the original dataset can be found in Table B in S1 File). Because of the observational nature of the study, it was not possible to establish control groups. Alternatively the survey aimed to collect information to account for all potential confounders that would allow to tease out the effect of park usage and accessibility on well-baing.

#### Explanatory variables considered

Four groups of explanatory variables were considered. Firstly, respondents were asked on their socioeconomic background, namely their age, gender and income per household capita. Secondly, the questionnaire sought to understand their state of health by asking about their level of physical activity and serious health problems (SI). Thirdly, the questionnaire asked about their personality traits of extraversion and neuroticism, or emotional stability. Extraversion is associated with energy, sociability and assertiveness, whereas emotional stability refers to the tendency to experience negative emotions such as anger and depression easily [34]. These traits were included into the study because of the presence of strong existing research evidence that suggested their influence on well-being [35]. Two relevant questions from the Ten-Item Personality Inventory [36] were used to measure extraversion, and another two to measure emotional stability (SI).

The fourth group of explanatory variables was the one of interest to this study. Here, the questionnaire asked respondents about their use and access of green spaces. Four types of green spaces were defined in this context, namely: (1) Nature reserves that are are sites of importance for wildlife, flora and fauna that are legally protected; (2) Regional parks that are larger parks with facilities that serve the wider region; (3) Neighbourhood parks that are smaller parks that serve the residential neighbourhood nearby; and (4) Park connectors that are linear corridors that link major parks, nature sites and population centres. They are typically located along drainage canals and roads.

For each of the four types of green space that were investigated, three measures were used in the survey questionnaire to measure the green space usage of respondents:

- the number of days that the respondents visited a particular type of green space *g* which lies near to their house in a year (*F*_*g(N)*_),
- the number of days that the respondents visited a particular type of green space which lies far from their house in a year (*F*_*g(F)*_), and
- the typical duration of visiting each type of green space (*D*_*g*_).

A green space was considered far from the respondent’s house if they could not get there within ten minutes on foot. The respondent's usage of each type of green space was then calculated according to Eq (1):

$$U_{g}=\sum_{g}^{n}F_{g(N)}\cdot D_{g}+F_{g(F)}\cdot D_{g}$$ (1)

where *U*_*g*_ was the respondent's usage of a particular type of green space in a year.

**Fig 2:**
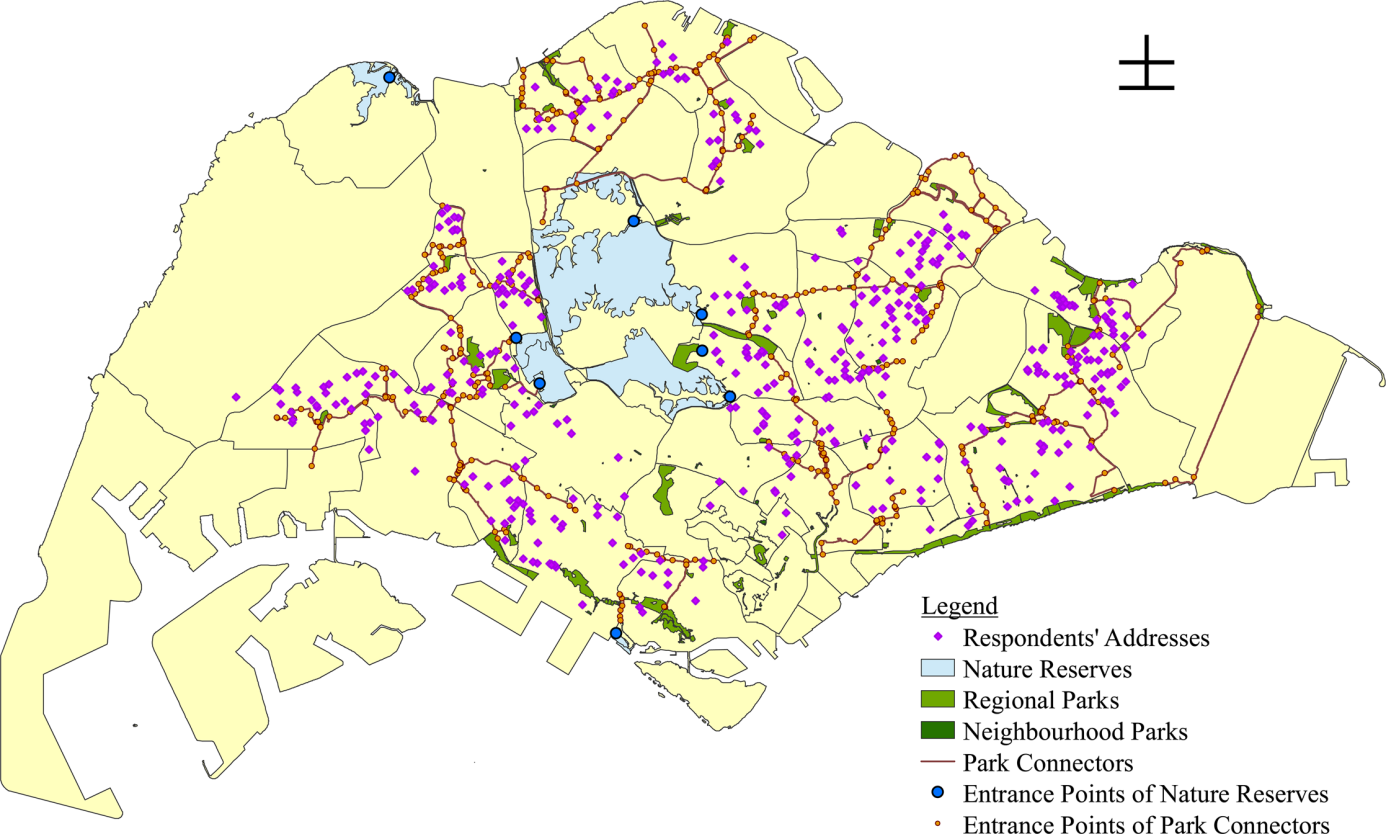
Locations of four types of green spaces in Singapore with respect to respondents’ addresses.

A measure for each respondent's access to different types of green space was also derived. Respondents' postal codes were mapped as points in ArcGIS 10.2.2, on top of separate polygon shapefiles of nature reserves, regional parks, neighbourhood parks and park connectors that were provided by the National Parks Board (Fig 2).

Each respondent's access to each type of green space was then calculated according to Eq (2):

$$A_{i}=\sum_{g}s^{ß}{}_{g}/d^{\lambda }{}_{ig}$$ (2)

*A*_*i*_ is the respondent's access index at his or her 6-digit Singapore postal code *i*, for a particular type of green space. *s*_*g*_ is the size of the green space in square metres, and *ß* is a size decay parameter. *d*_*ig*_ is the distance between the respondent's postal code *i* and the green space *g*, whereas *λ* is a distance-decay parameter between *i* and *g* [37].

ArcGIS was used to estimate the accessibility to green spaces within a Euclidean distance of 1.2 km from the respondent's postal code. For nature reserves, regional parks and neighbourhood parks, all green spaces within this 1.2km buffer were included in the summation equation. However, for park connectors, only the nearest park connector was included in the calculation of *A*_*i*_ because these connectors are likely to be interconnected given the existing park connector network in Singapore (Fig 2).

*s*_*g*_ was taken from the area data provided in NParks’ polygon shapefiles for nature reserves, regional parks and neighbourhood parks (Fig 2). However, for park connectors, their area was assumed to be universally 1ha to account for their linearity.

For regional parks and neighbourhood parks, *d*_*ig*_ was calculated as the distance between the respondent's residence *i* and the nearest point along the boundary of the green space *g*. However, for nature reserves and park connectors, *d*_*ig*_ was calculated as the distance between the respondent's residence *i* and the nearest entrance point of the green space *g*. This is because nature reserves and park connectors are only accessible through certain points (Fig 2).

#### Dependent variables considered

Five dependent variables were used in this study to indicate well-being (Fig 1). The first and primary variable was subjective well-being (SWB), a key concept that has been developed in the field of psychology to measure and quantify well-being. SWB adopts the hedonistic approach in studying well-being, and proposes that individuals seek to maximize pleasure and minimize displeasure[38]. SWB is commonly associated with happiness, and comprises three basic components: positive affect (PA), negative affect (NA) and life satisfaction. These components were the second, third and fourth variables analysed in the study. The first two components, positive affect and negative affect, are the affective or emotional aspects of SWB [39], whereas the last component, life satisfaction, is the cognitive aspect [40].

Positive affect refers to the experience of pleasant positive emotions [41], whereas negative affect reflects unpleasant negative emotions [42]. It has been suggested that low positive affect and high negative affect are respectively associated with depression and anxiety [41]. Studying these affective aspects in an individual can thus give an indication of mental health.

Life satisfaction, the cognitive aspect, refers to a subjective global self-assessment of one’s quality of life [40]. Here, it is key that this assessment is centred on criteria specific to the individual [38]. Because individuals place different value on their different domains of life, happiness as indicated by life satisfaction measurements tend to be based on the assessment of life as a whole [40].

SWB was measured using two items. The first was the Satisfaction with Life Scale, used to measure life satisfaction [40]. This consisted of five questions where participants rated on a scale from 1 to 7 how much they agreed with a given statement about their life satisfaction (SI). The second item was the Positive Affect and Negative Affect Scale. This scale consisted of ten questions, five of which measured positive affect and the remaining five measured negative affect [43] (SI). Respondents were asked to rate on a scale from 1 to 5 how often they generally felt a particular emotion. The aggregate score for SWB was calculated according to Eq (3) below:

SWB=Positive Affect−Negative Affect+Life Satisfaction (3)

A fifth and final dependent variable was included to supplement its negative affect component and thereby further indicate well-being. This dependent variable was perceived stress, which is a self-evaluation of one's stress in life [44]. Respondents' stress levels were measured using the shortened version of the Perceived Stress Scale [44]. This scale had four items that asked participants to rate on a scale from 1 to 5 how often they felt certain feelings about their lives in the past month (SI).

### Statistical analysis

#### Model Fitting

Generalized least squares (GLS) models were used to determine the relationship between SWB and both the access to different green spaces as well as the use of different green spaces. GLS modelling was chosen to account for possible spatial autocorrelation in the dataset. To gain a better understanding of the specific aspects of SWB affected, the same analysis procedure was conducted for the individual component variables of SWB as the dependent variable, namely positive affect, negative affect and life satisfaction. This analysis was also repeated for the perceived stress score as the dependent variable.

To find the best spatial autocorrelation structure for the model, we ran the saturated model multiple times, each time with a different spatial autocorrelation structure. The saturated model included all the variables considered in this study, where all non-categorical variables had been scaled so that their effect sizes were directly comparable. We then compared the resultant Akaike Information Criterion (AIC) values. The model without any spatial autocorrelation structure yielded the lowest AIC value. Thus, no spatial autocorrelation structure was used in the GLS models finally considered. This is equivalent to a multiple linear regression model. The validity of this model was confirmed by checking that the residuals did not present problems of heteroscedasticity and conformed to the normality assumption.

Next, the variables in the model were tested for multicollinearity by calculating their variance inflation factors (VIF) in a model containing the main effects of all the explanatory variables. The VIFs for all variables presented a VIF score below two, indicating that none of the variables were collinear.

#### Model Selection

Model selection was conducted using the information theoretic approach [45]. The information theoretic approach estimates parameters based on multimodal inference and recognizes that datasets can support multiple competing models and hypotheses [45]. This method was selected in this investigation due to the many potential plausible hypotheses that could affect well-being.

Model selection was based on *AIC*_*c*_, a variant of the AIC corrected for potential bias due to small samples [45]. 76 models, each with different combinations of variables, were run and the model with the smallest *AIC*_*c*_ value (*AIC*_*c*,*min*_) was taken as the best model. The *AIC*_*c*_ difference for each model *m*, *Δ*_*m*_, was computed as *AIC*_*c*,*m*_—*AIC*_*c*,*min*_ for all the candidate models run. Models with *Δ*_*m*_<2 were eventually included in the computation of the model average parameters. To quantify the relative plausibility of each model, the Akaike weight, *w*_*m*_, of each model was also calculated. The final model was then derived by computing the model average, taking into account the weight of each model in the set. This was achieved by using the MuMIn package in R.

We used the Bonferroni correction to correct for multiple comparisons [46]. Given that five dependent variables were considered, the cut-off for a significant p-value was taken to be 0.01.

## Results

The majority of the respondents belonged to the student population ranging from 18 to 25 years old, and almost all the respondents were unmarried and had no children. Table A in S1 File shows a descriptive summary of the survey respondents sampled.

**Fig 3:**
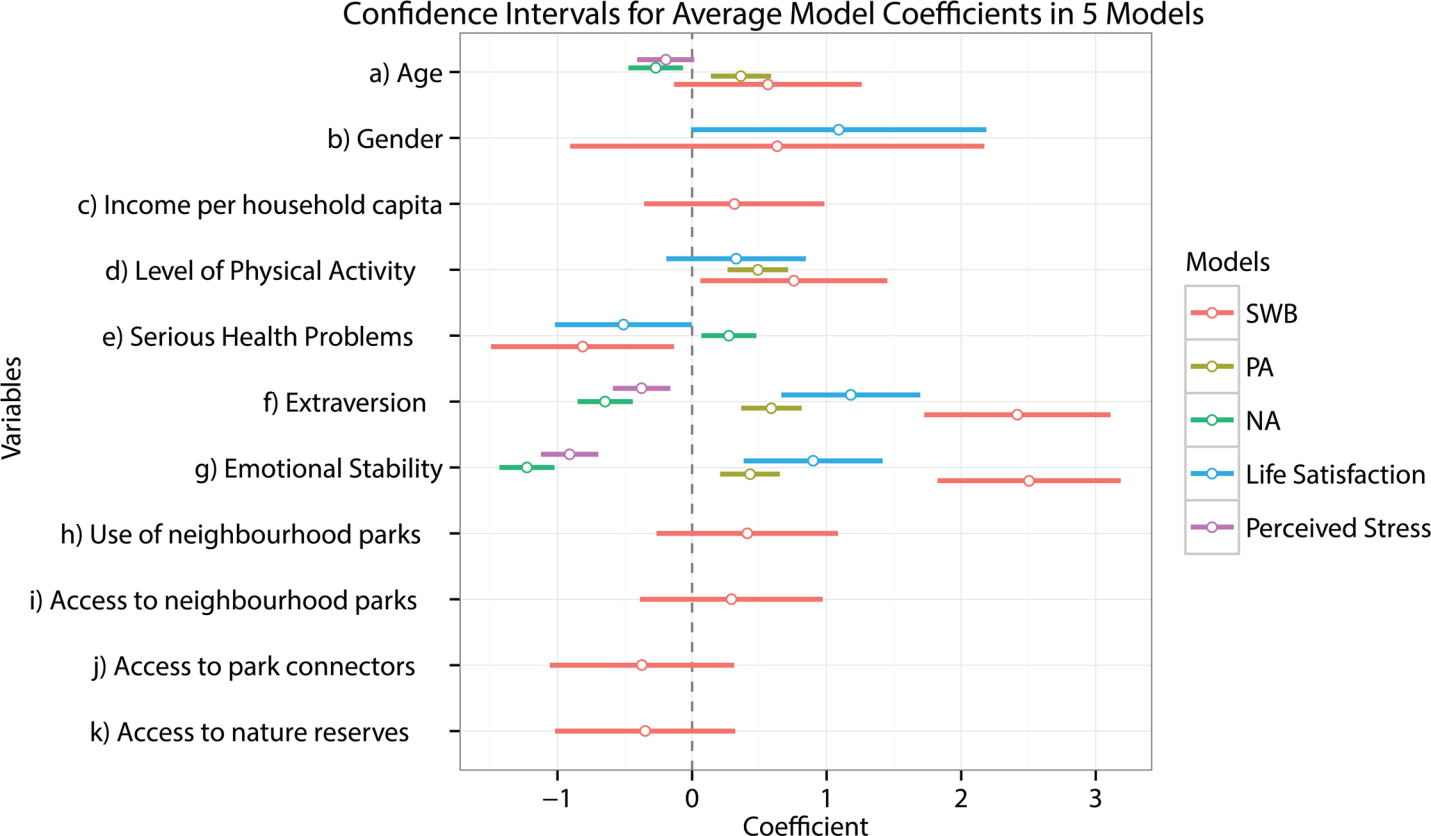
95% confidence intervals of variables in the average model coefficients for five models. The variable coefficients in the models for SWB, positive affect and life satisfaction have opposite signs from the coefficients of the same variables in the models for negative affect and perceived stress, if they appear in the model averages again. This is because negative affect and perceived stress are indications of negative well-being whereas SWB, positive affect and life satisfaction are positive indications of well-being.

The model average from the information theoretic approach showed no strong evidence that any green space variables significantly affected SWB, i.e. the confidence intervals of all the relevant green space variables overlapped with zero (Fig 3, p-values > 0.05, Table 1).

**Table 1:** Model-averaged coefficients of variables and their relative importance in the model for SWB. CI: confidence interval.

Variable	Model-averaged coefficients	CI 2.5%	CI 97.5%	p-value	Relative variable importance
Intercept	25.16	24.11	26.21	<0.001**	N.A.
Age	0.56	-0.13	1.26	0.11	0.54
Gender (Female)	0.64	-0.90	2.18	0.42	0.43
Household income per capita	0.31	-0.36	0.98	0.36	0.04
Level of Physical Activity	0.76	0.06	1.45	0.03*	0.96
Serious Health Problems	-0.82	-1.50	-0.14	0.02*	1.00
Extraversion	2.42	1.72	3.11	<0.001**	1.00
Emotional Stability	2.50	1.82	3.18	<0.001**	1.00
Use of neighbourhood parks	0.41	-0.26	1.08	0.23	0.21
Access to neighbourhood parks	0.29	-0.39	0.97	0.40	0.04
Access to park connectors	-0.37	-1.06	0.31	0.29	0.18
Access to nature reserves	-0.34	-1.01	0.34	0.33	0.16

** indicates a significance level of <0.01 (before Bonferroni correction) and

* indicates a significance level of <0.05 (before Bonferroni correction).

However, the model average showed two other key factors which significantly affect SWB—extraversion and emotional stability (Table 1). These personality attributes displayed the most dominant effects on SWB, showing large positive coefficient values (2.50 and 2.42 for emotional stability and extraversion respectively) that were highly significant (p-values <0.001).Both variables appeared in all the component models used to compute the model average (relative importance of 1.00 for both).

Two additional variables also had considerable effect on SWB, although they did not hit the p-value cut-off criteria after the Bonferroni correction. Level of physical activity positively affected SWB (coefficient = 0.76, p-value 0.03) and serious health problems negatively affected SWB (coefficient = -0.82, p-value 0.02). Serious health problems appeared in all the component models used to compute the model average (relative importance 1.00) whereas level of physical activity appeared in all the component models except one (relative importance 0.96), indicating their potential role in influencing SWB.

Although none of the 10 green space variables studied appeared to have a significant effect on SWB, there was variation among their relative importance to the model average. The use of neighbourhood parks appeared to be the most important in affecting SWB (relative importance 0.21) followed by, access to park connectors (relative importance 0.18), access to nature reserves (relative importance 0.16) and access to neighbourhood parks (relative importance 0.04). Use of neighbourhood parks (coefficient 0.41) and access to neighbourhood parks (coefficient 0.29) presented a positive relationship with SWB whereas access to park connectors (coefficient -0.37) and access to nature reserves (coefficient -0.34) had a negative relationship. Four other green space variables, namely, access to regional parks, use of regional parks, use of neighbourhood parks and use of park connectors, were used in the candidate model set, but were not included in the model average.

To look into specific aspects of well-being, four subsequent analyses were conducted with either positive affect, negative affect, life satisfaction or perceived stress as the dependent variables. The results showed that the model average for SWB included the greatest number of variables as compared to the model averages for the other dependent variables. Fig 3 shows the eleven variables that were used in the model average of the SWB model, yet out of these, only a few appeared in the model averages for the other four models.

Notably, in the four model averages other than the SWB model average, no green space variables were included at all. This suggests again that the use and access of green space do not significantly affect well-being. Instead, the most significant variables affecting the other four dependent variables were again extraversion and emotional stability. Extraversion and emotional stability produced strong positive effects on positive affect (coefficient 0.59 and 0.43 respectively) and life satisfaction (coefficient 1.18 and 0.90 respectively); however, as expected, they appeared to reduce negative affect (coefficient -0.65 and -1.23 respectively) and perceived stress (coefficient -0.38 and -0.91 respectively). The confidence intervals for the coefficients of these variables in the models for SWB, positive affect and life satisfaction lay clearly to the right of 0, whereas that for the models of negative affect and perceived stress lay clearly to the left (Fig 3). In all five models, the p-values for extraversion and emotional stability were always less than 0.001 (Tables 1, 2, 3, 4 and 5). These results thus underline the strong and significant effect that extraversion and emotional stability have on well-being in various aspects.

**Table 2:** Model-averaged coefficients of variables and their relative importance in the model for positive affect. The model averages for positive affect was computed only from one model because there was only one model with *Δ*_*m*_<2 (AICc_m_ was 1942.70). CI: confidence interval.

Variable	Model-averaged coefficients	CI 2.5%	CI 97.5%	p-value	Relative variable importance
Intercept	16.80	16.58	17.02	<0.001**	N.A.
Age	0.36	0.14	0.59	0.002**	1.00
Level of Physical Activity	0.49	0.26	0.71	<0.001**	1.00
Extraversion	0.59	0.36	0.81	<0.001**	1.00
Emotional Stability	0.43	0.21	0.65	<0.001**	1.00

** indicates a significance level of <0.01 (before Bonferroni correction) and

* indicates a significance level of <0.05 (before Bonferroni correction).

**Table 3:** Model-averaged coefficients of variables and their relative importance in the model for negative affect. The model averages for negative affect was computed only from one model because there was only one model with *Δ*_*m*_<2 (AICc_m_ was 1870.44). CI: confidence interval.

Variable	Model-averaged coefficients	CI 2.5%	CI 97.5%	p-value	Relative variable importance
Intercept	13.70	13.50	13.90	<0.001**	N.A.
Age	-0.27	-0.47	-0.07	0.009**	1.00
Serious Health Problems	0.27	0.07	0.48	0.009**	1.00
Extraversion	-0.65	-0.85	-0.44	<0.001**	1.00
Emotional Stability	-1.23	-1.43	-1.02	<0.001**	1.00

** indicates a significance level of <0.01 (before Bonferroni correction) and

* indicates a significance level of <0.05 (before Bonferroni correction).

**Table 4:** Model-averaged coefficients of variables and their relative importance in the model for Life Satisfaction. CI: confidence interval.

Variable	Model-averaged coefficients	CI 2.5%	CI 97.5%	p-value	Relative variable importance
Intercept	21.69	20.67	22.72	<0.001**	N.A.
Gender (Female)	1.09	-0.01	2.19	0.05	0.75
Level of Physical Activity	0.33	-0.20	0.85	0.22	0.29
Serious Health Problems	-0.51	-1.02	0.00	0.05*	0.54
Extraversion	1.18	0.66	1.69	<0.001**	1.00
Emotional Stability	0.90	0.38	1.42	<0.001**	1.00

** indicates a significance level of <0.01 (before Bonferroni correction) and

* indicates a significance level of <0.05 (before Bonferroni correction).

**Table 5:** Model-averaged coefficients of variables and their relative importance in the model for Perceived Stress. CI: confidence interval.

Variable	Model-averaged coefficients	CI 2.5%	CI 97.5%	p-value	Relative variable importance
Intercept	9.45	9.23	9.66	0.000**	N.A.
Age	-0.20	-0.41	0.02	**0.07**	0.34
Extraversion	-0.38	-0.59	-0.16	0.001**	1.00
Emotional Stability	-0.91	-1.12	-0.70	0.000**	1.00

** indicates a significance level of <0.01 (before Bonferroni correction) and

* indicates a significance level of <0.05 (before Bonferroni correction).

Personality traits were the only two variables that significantly affected perceived stress. However, other factors further significantly affected positive affect, negative affect and life satisfaction (see Fig 3). Serious health problems increased negative affect (coefficient 0.27, p-value 0.009), which was expected. An increase in physical activity significantly increased positive affect (coefficient 0.49, p-value <0.001), suggesting that physical activity makes people feel good. Lastly, age significantly increased positive affect (coefficient 0.36, p-value 0.002) and reduced negative affect (coefficient -0.27, p-value 0.009), suggesting that an increase in the age of students tends to produce overall improvements in their mood. The remaining variables such as gender and household income per capita were not significant predictors of any of the five well-being indicators at all, having high p-values (>0.05) in all models.

## Discussion

This study found no significant relationship between well-being and use of green space as well as proximity to green spaces. Among the five models with different dependent variables, green space variables appeared only in the model average for the SWB model. A statistically not significant positive relationship was found between access to neighbourhood parks and SWB with a small variable importance of 0.04, and not significant negative relationships were also reported between well-being and the access to park connectors and nature reserves. The sole green space use variable that appeared in the model average for SWB was the use of neighbourhood parks. It had a positive relationship with SWB, but this relationship was also not statistically significant. Even when all five model analyses were repeated using an aggregate variable for green space use and another aggregate variable for green space access, each of which combined the four types of green spaces, they still remained not significant (Tables E and F in S1 File). Hence, this study suggests a lack of a significant relationship between well-being and access to green spaces as well as use of green spaces in Singapore.

This contradicts numerous previous findings that suggest a significant positive relationship between access or use of green space and well-being. For example, Van den Berg, Maas (8] showed that green spaces can alleviate stress and MacKerron and Mourato (9] demonstrated that natural environments produced greater happiness. Yet, the discrepancy in this study’s results cannot be attributed simply to issues with the research methodology, given that the sample size of this study, 426, is moderately large in comparison to other well-being studies with sample sizes ranging from 102 [47] to 1108 [48]. Hence, it is necessary to examine the points of divergence between the conditions of this study and those of previous studies to understand why there is a difference in the results obtained.

Firstly, the study represents an urban-level investigation that has collected data from residents who live across Singapore. Hence, the results of this study represent aggregate-level findings that generalize the relationship between well-being and the use or access of green spaces on the scale of the city. In contrast, many influential papers that have advocated the benefits of green spaces on well-being were conducted on specific groups of people within a city [49–52]. For example, studies conducted by Ulrich [49], Ulrich and Parsons [53] and White and Heerwagen [52] suggested that views of nature had restorative influences on sick patients within healthcare venues. Moreover, Moore [50] and West [51] also showed that natural views lowered the need to visit healthcare facilities for inmates in prisons. The results of these studies tend to be more relevant to particular physical settings that are of a smaller scale than the city, such as hospitals or prisons. On the aggregate urban scale, however, these effects may not necessarily still be detectable. There may also have been features or qualities specific to hospital patients or prison inmates that contributed to the positive relationship between access to green spaces and well-being in these studies, but which do not apply as strongly to the wider urban environment or the young adult group studied. Thus, this potentially explains why this study has failed to support the association between well-being and the use and access of green spaces, as has been traditionally suggested by environmental psychologists.

Secondly, many previous urban research studies on the relationship between the use of green space and well-being have been conducted in temperate regions such as Europe. For example, in the United Kingdom, it was reported that individuals living in urban areas with higher green space provision scored better on well-being [10]. Also, in Swedish cities, higher frequencies of visiting urban green spaces were associated with fewer stress-related illnesses [54]. However, there is a dearth of research conducted in the tropical climate where Singapore lies. Previous studies in Europe have indicated that green spaces can ameliorate urban heat [55, 56], hence, one may feel cooler in green spaces than in other spaces within the urban matrix. Although green spaces in Singapore similarly help to lower ambient temperatures of the urban environment [57], Singapore differs from Europe in that it heavily relies on air-conditioning to cool its buildings in its perpetually hot and humid climate. This tends to drive people indoors to enjoy thermal relief, thereby subverting the cooling benefits that green spaces offer. Hence, climatic differences may explain the variation in the quality of one’s experience when using urban green spaces within different parts of the world. Singapore’s unique climate and reliance on air-conditioning may be a reason why the results of this study indicate that the use of green spaces does not significantly improve well-being, contradicting studies conducted in temperate regions.

Thirdly, Singapore’s provision of urban greenery is higher than most other cities. For a global city, it offers one of the world's highest percentages of public green space at 47%, in comparison to New York (14%), London (38.4%) and Hong Kong (41%) [58]. Hence, the population may not vary sufficiently in terms of proximity to green spaces to demonstrate that access to green spaces has a significant effect on SWB. The relatively high pervasion of greenery on the island may cause the fatigue alleviation effect of green spaces, as according to the Attention Restoration Theory [12], to occur consistently for people throughout the city. People may easily feel refreshed and restored by the surrounding greenery without the need to be sited within a green space with predefined boundaries. Therefore, the results of this study do not necessarily imply that the use of green spaces or access to green spaces fail to significantly improve well-being. Instead, it may imply that the well-being benefits of green spaces become difficult to detect on the city level in the context of Singapore, even if they apply on the individual level. This is attributable to Weber’s Law, which states that a greater change in stimulus is needed for it to be noticeable if the original stimulus was of higher intensity. Thus, this can result in a phenomenon whereby increased access to green spaces or increased use of green spaces no longer produce a detectable significant difference on well-being because of the already high pervasion of greenery in the city. This potentially points to the relatively even distribution of greenery in Singapore, suggesting social equality in terms of people’s benefits from urban green spaces.

Fourthly, Singapore has rich biodiversity despite its small size. The ability of species such as birds to move freely across the small island may allow people to experience biodiversity even if they are not physically situated within green spaces. Singapore is home to 384 species of birds in its 710 km^2^ of land area [59, 60]. In comparison, Hong Kong’s land area is bigger than Singapore’s by 56% [58], but its bird species count is higher only by 38% [61]. Similarly, New York’s land area is bigger by 71% [58], but its bird species count is higher only by 24% [62], and London has a land area greater than Singapore’s by 121%, [58] but a bird species number greater only by 56% [63]. Thus, Singapore’s higher biodiversity relative to its land area may allow people more opportunities to come into contact with wildlife on a regular basis. This may explain why the effects of affiliating with nature, as suggested by the biophilia hypothesis [16], becomes more difficult to detect.

The most statistically significant finding of this study was that extraversion and emotional stability strongly affected well-being to a large extent. Specifically, extraversion had the greatest influence over positive affect and emotional stability had the greatest influence over negative affect. This supports existing literature which state that these two personality traits are important in predicting well-being [64]. Existing literature also state that extraversion primarily influences positive affect whereas emotional stability primarily influences negative affect [65, 66]. Hence, this suggests that well-being studies should account for the influences of extraversion and emotional stability when comparing the effects of using green spaces on well-being between different individuals. Otherwise, it will be difficult to ascertain if the reported well-being improvements are a result of variation in the use of green spaces between individuals, or a result of the personality differences that have been overlooked.

The results also illustrated that serious health problems significantly increased negative affect and that physical activity significantly increased positive affect. This is consistent with the dominant research literature such as Gerdtham & Johannesson [67] and Rascuite & Downward [68], and reinforces the importance of health on different aspects of one’s well-being.

This study only sampled the student population in the National University of Singapore, which mainly comprised of educated and unmarried adults aged 25 and below. Hence, this sample population may be non-representative of the general population [69]. The patterns of using green space for diverse sample populations and the factors significantly affecting well-being may vary due to changing life preferences and circumstances, and this may thereby affect the relative effect of using green spaces on well-being. For example, the mean frequency of park visits in Hong Kong was higher for married couples and the elderly compared to other demographic groups [70]. Although it is not uncommon to use university students as participants in similar research studies [15, 71], it will be good to test the applicability of these findings to other population groups to test whether these results hold in other age groups. This will ascertain that the lack of a relationship between the use of green spaces and well-being is not merely a result of specific factors that pertain only to the student population.

Unlike most previous research, this study did not find a significant relationship between well-being and the use or access of green space on the aggregate-level. This suggests that such a relationship is in fact contingent on other factors, such as urban climate, land area and greenery coverage. Hence, this study reveals that without first considering the critical factors that permit this relationship to occur, it will be premature to conclude that an increase in green space provision will lead to a direct increase in well-being. The existing research studies in this field tend to be relatively homogeneous in their setting, being based mostly in Europe or North America. Hence, this gives rise to many underlying questions about the conditions which result in the significant relationship between well-being and use of green space.

## Supporting Information

S1 File Supporting Tables A-F and Survey A. (DOCX)

S2 File Original Dataset. (XLSX)
