# Supplementary material for: The Relationship between Natural Park Usage and Happiness Does Not Hold in a Tropical City-State
Source: PLoS One. 2015 Jul 29;10(7):e0133781. doi: 10.1371/journal.pone.0133781 (PMC4519055; doi:10.1371/journal.pone.0133781)
Supplement: S1 File — (DOCX) [file pone.0133781.s001.docx]

**The relationship between natural park usage and happiness does not hold in a tropical city-state**

L.E. Saw^1^, F.K. Lim^1^, L.R. Carrasco^1,*^

^1^Department of Biological Sciences, National University of Singapore,14 Science Drive 4, Singapore 117543, Republic of Singapore.

^*^Email: dbsctlr@nus.edu.sg. Tel: +6591377291. Fax: +65 67792486.

**Supporting Tables A-F and Survey A.**

**Table A. Description of survey respondents.**

| Characteristic | Percentage of Sample (n = 426) |
| --- | --- |
| **Age (years)** |  |
| 18 – 20 | 52.8 |
| 21 – 25 | 45.3 |
| 26 – 30 | 1.41 |
| 31 - 35 | 0.47 |
| **Gender** |  |
| Male | 32.0 |
| Female | 68.0 |
| **Marital Status** |  |
| Unmarried | 99.8 |
| Married | 0.2 |
| **Number of children** |  |
| 0 | 99.8 |
| 1 | 0.2 |
| **Employment Status** |  |
| Unemployed | 91.5 |
| Part-time | 7.3 |
| Full-time | 1.2 |
| **Monthly household income ($)** |  |
| 0 – 5,000 | 35.4 |
| 5,000 – 10,000 | 40.8 |
| 10,000 – 15,000 | 13.4 |
| 15,000 – 20,000 | 5.2 |
| 20,000 – 25,000 | 2.35 |
| 25,000 – 30,000 | 0.7 |
| 30,000 – 35,000 | 1.2 |
| Above 35,000 | 0.8 |
| **Visits green spaces at least once a year** |  |
| Any green space | 89.0 |
| Nature reserves | 59.9 |
| Regional parks | 78.4 |
| Neighbourhood parks | 73.2 |
| Park connectors | 66.9 |

**Table B. Component models in the model average for SWB for *Δ_m_*< 2.** Variables included are age (AGE), gender (GEN), household income per capita [1], level of physical activity (PHY), serious health problems (HEA), extraversion (EXV), emotional stability (EMS), use of neighbourhood parks (UNP), access to neighbourhood parks (ANP), access to park connectors (APC) and access to nature reserves (ANR).

| Model | Df | AICc_m_ | Delta (*Δ_m_)* | Weight |
| --- | --- | --- | --- | --- |
| [PHY, HEA, EXV, EMS] | 6 | 2876.67 | 0.00 | 0.09 |
| [AGE, GEN, PHY, HEA, EXV, EMS] | 8 | 2876.69 | 0.02 | 0.09 |
| [AGE, PHY, HEA, EXV, EMS] | 7 | 2876.87 | 0.20 | 0.08 |
| [GEN, PHY, HEA, EXV, EMS] | 7 | 2877.11 | 0.44 | 0.07 |
| [PHY, HEA, EXV, EMS, UNP] | 7 | 2877.53 | 0.87 | 0.06 |
| [AGE, GEN, PHY, HEA, EXV, EMS, APC] | 9 | 2877.58 | 0.91 | 0.06 |
| [AGE, GEN, PHY, HEA, EXV, EMS, UNP] | 9 | 2877.72 | 1.05 | 0.05 |
| [AGE, PHY, HEA, EXV, EMS, APC] | 8 | 2877.88 | 1.21 | 0.05 |
| [GEN, PHY, HEA, EXV, EMS, UNP] | 8 | 2877.89 | 1.22 | 0.05 |
| [AGE, PHY, HEA, EXV, EMS, UNP] | 8 | 2877.99 | 1.32 | 0.05 |
| [AGE, GEN, PHY, HEA, EXV, EMS, ANR] | 9 | 2878.01 | 1.35 | 0.05 |
| [PHY, HEA, EXV, EMS, ANR] | 7 | 2878.17 | 1.50 | 0.04 |
| [PHY, HEA, EXV, EMS, APC] | 7 | 2878.20 | 1.53 | 0.04 |
| [INC, PHY, HEA, EXV, EMS] | 7 | 2878.20 | 1.53 | 0.04 |
| [AGE, PHY, HEA, EXV, EMS, ANR] | 8 | 2878.27 | 1.61 | 0.04 |
| [AGE, HEA, EXV, EMS] | 6 | 2878.46 | 1.79 | 0.04 |
| [AGE, PHY, HEA, EXV, EMS, ANP] | 8 | 2878.52 | 1.85 | 0.04 |
| [GEN, PHY, HEA, EXV, EMS, ANR] | 8 | 2878.58 | 1.91 | 0.03 |
| [GEN, PHY, HEA, EXV, EMS, APC] | 8 | 2878.63 | 1.97 | 0.03 |

**Table C. Component Models in the Model Average for Life Satisfaction for *Δ_m_*< 2.** Variables included are gender (GEN), level of physical activity (PHY), serious health problems (HEA), extraversion (EXV), emotional stability (EMS) and exercise in green spaces (GEX).

| Model | Df | AICc_m_ | Delta**(***Δ_m_)* | Weight |
| --- | --- | --- | --- | --- |
| [GEN, EXV, EMS] | 10 | 2631.89 | 0.00 | 0.46 |
| [GEN, PHY, HEA, EXV, EMS] | 7 | 2632.82 | 0.93 | 0.29 |
| [HEA, EXV, EMS] | 5 | 2633.11 | 1.22 | 0.25 |

**Table D. Component Models in the Model Average for Perceived Stress for *Δ_m_*< 2.** Variables included are age (AGE), extraversion (EXV) and emotional stability (EMS). ** indicates a significance level of <0.01 (after Bonferroni correction) and * indicates a significance level of <0.05 (before Bonferroni correction).

| Model | Df | AICc_m_ | Delta**(***Δ_m_)* | Weight |
| --- | --- | --- | --- | --- |
| [EXV, EMS] | 4 | 1900.74 | 0.00 | 0.66 |
| [AGE, EXV, EMS] | 5 | 1902.10 | 1.37 | 0.34 |

**Table E. Model-averaged coefficients of variables and their relative importance in the model for SWB, when aggregate green space use and access variables are used instead of individual-type green space variables.** ** indicates a significance level of <0.01 (after Bonferroni correction) and * indicates a significance level of <0.05 (before Bonferroni correction).

| Variable | Model-averaged coefficients | Confidence Interval | | p-value | Relative variable importance |
| --- | --- | --- | --- | --- | --- |
|  |  | 2.5% | 97.5% |  |  |
| (Intercept) | 25.18 | 24.17 | 26.20 | <0.001** | N.A. |
| Age | 0.56 | -0.13 | 1.25 | 0.11 | 0.50 |
| Gender (Female) | 0.61 | -0.92 | 2.15 | 0.43 | 0.39 |
| Household income per capita | 0.31 | -0.36 | 0.98 | 0.36 | 0.10 |
| Level of Physical Activity | 0.76 | 0.07 | 1.45 | 0.03* | 0.91 |
| Serious Health Problems | -0.80 | -1.48 | -0.13 | 0.02* | 1.00 |
| Extraversion | 2.42 | 1.73 | 3.11 | <0.001** | 1.00 |
| Emotional Stability | 2.51 | 1.83 | 3.19 | <0.001** | 1.00 |

**Table F. Component models in the model average for SWB that uses aggregate greenspace use and access variables, for *Δ_m_*< 2.** Variables included are age (AGE), gender (GEN), household income per capita [1], level of physical activity (PHY), serious health problems (HEA), extraversion (EXV), emotional stability (EMS), use of neighbourhood parks (UNP), access to neighbourhood parks (ANP), access to park connectors (APC) and access to nature reserves (ANR).

| Model | Df | AICc_m_ | Delta (*Δ_m_)* | Weight |
| --- | --- | --- | --- | --- |
| [PHY, HEA, EXV, EMS] | 6 | 2876.67 | 0.00 | 0.22 |
| [AGE, GEN, PHY, HEA, EXV, EMS] | 8 | 2876.69 | 0.02 | 0.22 |
| [AGE, PHY, HEA, EXV, EMS] | 7 | 2876.87 | 0.20 | 0.20 |
| [GEN, PHY, HEA, EXV, EMS] | 7 | 2877.11 | 0.44 | 0.18 |
| [INC, PHY, HEA, EXV, EMS] | 7 | 2878.20 | 1.53 | 0.10 |
| [AGE, HEA, EXV, EMS] | 6 | 2878.46 | 1.79 | 0.08 |

**Survey A. Use of Green Spaces and Well-being of Participants.**

**Participant Information Sheet**

Research Protocol Title:

**Relationship between well-being and the access to and use of green space**

Principal Investigator:

**Dr. Roman Carrasco, NUS Dept of Biological Sciences, Faculty of Science**

**Tel: 66011145 or email: dbsctlr@nus.edu.sg**

Dear Student,

You are invited to participate in a research project titled above. This is a final year research study of a NUS Year 4 undergraduate student who majors in Environmental Studies. The aim of this research is to study the **relationship between using green spaces and the happiness of residents** who live in Singapore and are aged 18 years old and above (or 21 years old and above for non-NUS students).

This will involve **15 minutes** of your time to complete an anonymous survey that starts on page 2.

Apart from some basic demographic information, no personal, identifiable information will be obtained from you. You may refuse to answer any question if you are not comfortable.

Your decision to participate in this research is entirely voluntary and completely up to you. **Participation in this research will not affect your module grade in any way.** The voluntary completion of the survey will serve as your implied consent to participation.

Whom should I contact if I have any questions or problems?

1. You may contact **Ms. Saw Le En**, the student co-investigator if you have any queries regarding the research at 96890366 or email her at [le_en.saw@nus.edu.sg](mailto:le_en.saw@nus.edu.sg)) OR the Principal Investigator, **Dr. Roman Carrasco** (66011145 or [dbsctlr@nus.edu.sg](mailto:dbsctlr@nus.edu.sg)).
2. For an independent opinion regarding the research, you may contact a staff member of the National University of Singapore Institutional Review Board (Attn: **Mr. Chan Tuck Wai**, at 65-6516 1234 or [irb@nus.edu.sg](mailto:irb@nus.edu.sg)).

If you do not wish to participate in this research, kindly return the blank survey questionnaire.

If you have completed this survey questionnaire before, please do not participate again.

*There are 3 sections in this survey, 8 pages in total. Section I seeks basic information about you, Section II seeks to find out about your use of green spaces, and Section III seeks to find out how happy you are.*

**SECTION I**

*This section seeks some unidentifiable personal information to account for other variables that might influence your happiness.*

1. How old are you?

- Below 21
- 21-25
- 26-30
- 31-35
- 36-40
- 41-44

1. What is your gender?

- Male
- Female

1. What is your marital status?

- Single
- Married
- Widowed
- Separated
- Divorced

1. Are you currently employed?

- Yes, full time
- Yes, part time or temporary
- No, unemployed and looking for job
- No, unemployed but not looking for job
- No, student

1. How many children do you have?

- 0
- 1
- 2
- 3
- 4
- Others: _______(Please state)

1. How many other family members currently stay in your household?

- 0
- 1
- 2
- 3
- 4
- 5
- 6
- Others: _______(Please state)

1. What is your postal code number? (Please fill in with digits only)

*This question investigates the proximity of your residence to green space. The information obtained will not be used to identify you.*

S

1. What is the total income of your household per month?

- Less than $5000
- $5000 to $10,000
- $10,000 to $15,000
- $15,000 to $20,000
- $20,000 to $25,000
- $25,000 to $30,000
- $30,000 to $35,000
- $35,000 to $40,000
- $40,000 to $45,000
- $45,000 to $50,000
- Others (please state in thousands of dollars):

$_______________ to $_______________

1. What is the highest level of education that you have completed?

- Pre-primary school
- Primary school
- Secondary school
- ITE
- Polytechnic
- Junior College
- Accredited diploma
- University
- Masters or PhD

1. How often do you take part in physical activity?

(Each session should either be at least 30 min of moderately intensive activity or 20 min of intensive physical activity.)

- Less than once a week
- Once a week
- 2 times a week
- 3 times a week
- 4 times a week
- 5 times a week
- 6 times a week
- 7 times a week
- More than 7 times a week

1. To what extent have serious health problems impacted your quality of life? Please tick on a scale from 1 to 5:

*Somewhat*

3

*Slightly*

2

*Not at all*

1

*Very heavily*

5

*Heavily*

4

1. Below are a number of personality traits that may or may not apply to you. Please write a number next to each statement to indicate the extent to which you agree or disagree with it. You should rate the extent that the pair of traits applies to you, even if one characteristic applies more strongly than the other.

| Disagree strongly | Disagree moderately | Disagree a little | Neither agree nor disagree | Agree a little | Agree moderately | Agree strongly |
| --- | --- | --- | --- | --- | --- | --- |
| 1 | 2 | 3 | 4 | 5 | 6 | 7 |
| I see myself as:   1. _______ Extraverted, enthusiastic. 2. _______ Anxious, easily upset. 3. _______ Reserved, quiet. 4. _______ Calm, emotionally stable. | | | | | | |

**This is the end of Section I.**

**SECTION II**

*This section will focus on the pattern of your use of green spaces. In this survey, green spaces shall refer only to* ***nature reserves, regional parks, neighbourhood parks and park connectors in Singapore*** *that are open to the public.*

| *Nature Reserves (NR)* | *These are nature areas where flora and fauna are protected. Examples include Sungei Buloh Wetland Reserve, Labrador NR and Central Catchment NR.* |
| --- | --- |
| *Regional Parks* | *These are typically large parks with facilities for the wider population. Examples include Bishan-Ang Mo Kio Park, East Coast Park and Coney Island.* |
| *Neighbourhood Parks* | *These are smaller parks that serve the immediate residential community in HDB or private estates. Examples include Ann Siang Hill Park and Bishan Active Park.* |
| *Park Connectors (PC)* | *These are green corridors that link major parks and nature sites, and are typically located along drainage canals and roads. Examples include Bukit Panjang PC.* |

*For more examples of all four terms, please refer to Appendix I-IV.*

1. How frequently do you visit green spaces that are **within walking distance to your house**? (Eg. You get there by foot, in 10min or less. Please tick one option in each column, from a to d.)

|  | a) Nature Reserves | b) Regional Parks | c) Neighbourhood Parks | d) Park Connectors |
| --- | --- | --- | --- | --- |
| Daily |  |  |  |  |
| 3 times a week |  |  |  |  |
| Weekly |  |  |  |  |
| Monthly |  |  |  |  |
| Once in 3 months |  |  |  |  |
| Once in 6 months |  |  |  |  |
| Yearly |  |  |  |  |
| Never |  |  |  |  |

1. How frequently do you visit green spaces that are **far from your house**? (Eg. You cannot get there within 10 min by foot. Please tick one option in each column, from a to d.)

|  | a) Nature Reserves | b) Regional Parks | c) Neighbourhood Parks | d) Park Connectors |
| --- | --- | --- | --- | --- |
| Daily |  |  |  |  |
| 3 times a week |  |  |  |  |
| Weekly |  |  |  |  |
| Monthly |  |  |  |  |
| Once in 3 months |  |  |  |  |
| Once in 6 months |  |  |  |  |
| Yearly |  |  |  |  |
| Never |  |  |  |  |

If you ticked Never or N.A. for all the columns (a to d) in both Q13 and Q14, please skip to Q22 (pg 6).

1. How long do you normally spend in green spaces each time you visit them? (Please tick one option in each column, from a to d.)

|  | a) Nature Reserves | b) Regional Parks | c) Neighbourhood Parks | d) Park Connectors |
| --- | --- | --- | --- | --- |
| Less than 30 min |  |  |  |  |
| 30 min to 1 hour |  |  |  |  |
| 1 to 2 hours |  |  |  |  |
| 2 to 3 hours |  |  |  |  |
| 3 to 4 hours |  |  |  |  |
| Others  (Please state in column) |  |  |  |  |

1. How do you normally travel to green spaces when you visit them? (Please tick all that apply)

- Car
- Public transport
- Bicycle
- Walking

1. Who do you normally visit green spaces with? (Please tick all that apply)

- Alone
- Strangers
- Acquaintances
- Friends
- Distant family members
- Immediate family members

1. What is your purpose in visiting green spaces? (Please tick all that apply)

- Wildlife observation
- Visiting with pets
- Visiting with children
- Exercising
- Social activities
- Passing by on my way to elsewhere
- Passive enjoyment (eg. resting, meditation)
- Therapy (eg. visiting nature areas as recommended by doctors)
- Other (Please state): ___________________________

1. Do you prefer to visit certain types of green spaces over others?

- Yes
- No preference. I like all green spaces equally. (Please skip to q22.)
- I don't like to visit any green space at all. (Please skip to q22.)

1. Which green space do you prefer to visit? Please rank the following accordingly by writing 1 to 4 in the column provided (with 1 being the least preferred and 4 being the most preferred):

| Nature reserves |  |
| --- | --- |
| Regional Parks |  |
| Neighbourhood Parks |  |
| Park Connectors |  |

**This is the end of Section II.**

**SECTION III**

*This section measures the level of your well-being.*

1. Below are five statements which you may agree or disagree with. Please tick your response for each statement below, on a scale of 1 to 7:

|  | 1  Strongly disagree | 2  Disagree | 3  Slightly disagree | 4  Neither agree nor disagree | 5  Slightly agree | 6  Agree | 7  Strongly agree |
| --- | --- | --- | --- | --- | --- | --- | --- |
| 1. In most ways my life is close to my ideal |  |  |  |  |  |  |  |
| 1. The conditions of my life are excellent |  |  |  |  |  |  |  |
| 1. I am satisfied with my life |  |  |  |  |  |  |  |
| 1. So far I have gotten the important things I want in life |  |  |  |  |  |  |  |
| 1. If I could live my life over again, I would change almost nothing |  |  |  |  |  |  |  |

1. Thinking about yourself and how you normally feel, please tick your response to each row on a scale from 1 to 5:

|  | To what extent do you generally feel: | 1  Never | 2  Almost Never | 3  Sometimes | 4  Often | 5  Always |
| --- | --- | --- | --- | --- | --- | --- |
| a | Upset |  |  |  |  |  |
| b | Hostile |  |  |  |  |  |
| c | Alert |  |  |  |  |  |
| d | Ashamed |  |  |  |  |  |
| e | Inspired |  |  |  |  |  |
| f | Nervous |  |  |  |  |  |
| g | Determined |  |  |  |  |  |
| h | Attentive |  |  |  |  |  |
| i | Afraid |  |  |  |  |  |
| j | Active |  |  |  |  |  |

1. The questions below ask about your thoughts and feelings in the past month. Please tick your response to each row on a scale from 1 to 5:

|  | In the past month, how often have you: | 1  Never | 2  Almost Never | 3  Sometimes | 4  Fairly Often | 5  Often |
| --- | --- | --- | --- | --- | --- | --- |
| a | Felt that you were unable to control the important things in your life? |  |  |  |  |  |
| b | Felt confident about your ability to handle your personal problems? |  |  |  |  |  |
| c | Felt that things were going your way? |  |  |  |  |  |
| d | Felt difficulties were piling up so high that you could not overcome them? |  |  |  |  |  |

**This is the end of Section III and the end of the survey. Thank you very much for your participation!**

**Appendix I**

**List of Nature Reserves in Singapore**

*Nature reserves include Central Catchment Nature Reserve, Bukit Timah Nature Reserve, Labrador Nature Reserve and Sungei Buloh Wetland Reserve, as well as their associated parks only:*

1. Labrador Nature Reserve
2. Sungei Buloh Wetland Reserve
   1. Kranji Nature Trail
3. Bukit Timah Nature Reserve
   1. Hindhede Nature Park
   2. Dairy Farm Nature Park
4. Central Catchment Nature Reserve
   1. MacRitchie Reservoir Park
   2. Lower Peirce Reservoir Park
   3. Upper Peirce Reservoir Park
   4. Upper Seletar Reservoir Park

**Appendix II**

**Examples of Regional Parks in Singapore**

| Admiralty Park | HortPark | Springleaf Park |
| --- | --- | --- |
| Ang Mo Kio Town Garden East | Istana Park | Sun Plaza Park |
| Ang Mo Kio Town Garden West | Jurong Central Park | Tampines Bike Trail |
| Bedok Reservoir Park | Kallang Riverside Park | Tampines Eco Green Park |
| Bedok Town Park | Kent Ridge Park | Telok Blangah Hill Park |
| Bishan-Ang Mo Kio Park | Lower Seletar Reservoir Park | Tiong Bahru Park |
| Bukit Batok Nature Park | Marina Promenade | Toa Payoh Town Park |
| Bukit Batok Town Park  (Little Guilin) | Mount Faber Park | War Memorial Park |
|  | One-north Park | West Coast Park |
| Changi Beach Park | Pasir Ris Park | Windsor Interim Green |
| Choa Chu Kang Park | Pasir Ris Town Park | Woodlands Town Garden |
| Clementi Woods Park | Pearl's Hill City Park | Woodlands Waterfront |
| Dhoby Ghaut Green | Punggol Park | Yishun Neighbourhood Park |
| Duxton Plain Park | Punggol Waterway Park | Yishun Park |
| East Coast Park | Sembawang Park | Zhenghua Park |
| Esplanade Park | Sengkang Riverside Park |  |
| Fort Canning Park | Singapore Botanic Gardens |  |

**Appendix III**

**Examples of Neighbourhood Parks in Singapore**

| Aljunied Park | Holland Village Park | Stamford Green |
| --- | --- | --- |
| Ann Siang Hill Park | Kampong Glam Park | Sunset Way Park |
| Arthur Park | Kampong Java Park | Surin Avenue Neighbourhood Park |
| Bougainvillea Park | Kim Seng Park | Tanjong Pagar RICOH Park |
| Changi Point Waterfront Park | Malcolm Park | Tavistock Avenue Park |
| Chartwell Drive Park | Mount Emily Park | Telok Kurau Park |
| Chiltern Drive Interim Park | Namly Park | Thomson Park |
| Duchess Park | Novena Park | Turnhosue Park |
| East Coast Terrace Park | Novena Rise Park | Villa Verde Park |
| Elite Terrace Park | Parry Avenue Interim Park | Wilkinson Interim Park |
| Empress Place | Pasir Panjang Park | Yishun Pond Park |
| Fuyong Interim Park | Raffles Place Park | Youth Olympic Park |
| Gelenggang Park | Richards Avenue Park | Yunnan Park |
| Geylang East Park | Springside Park |  |

**Appendix IV**

**Examples of Park Connectors in Singapore**

| Admiralty West PC | Geylang PC | Sembawang PC |
| --- | --- | --- |
| Alexandra PC | Henderson PC | Serangoon PC |
| Ang Mo Kio Avenue 5 PC | Hillview PC | Siglap PC |
| Ang Mo Kio PC | Jalan Pelikat PC | Siglap-Kallang Basin PC Loop |
| Balam PC | Jurong PC | Simei PC |
| Bedok PC | Jurong West PC | Simpang Kiri PC |
| Buangkok PC | Kallang PC | Sungei Serangoon PC |
| Bukit Batok East PC | Khatib Bongsu PC | Tampines PC |
| Bukit Batok PC | Loyang PC | Tampines-Loyang PC |
| Bukit Batok West PC | Mandai PC | Ulu Pandan PC |
| Bukit Panjang PC | Marsiling PC | Ulu Sembawang PC |
| Bukit Timah PC | Pandan Gardens PC | West Coast PC |
| Canberra PC | Pang Sua PC | Whampoa PC |
| Canberra-Sembawang PC | Pasir Ris PC | Woodlands PC |
| Central Catchment PC | Paya Lebar PC | Yishun PC |
| Chua Chu Kang North PC | Pelton Canal PC |  |
| Chua Chu Kang PC | Punggol PC |  |

**References**

1. The New York State Ornithological Association Inc. (2014) Checklist of the Birds of New York State.

2. Streetdirectory (2010) HSBC Tree Top Walk - Macritchie Nature Trail.

3. Bingham-Hall P (2012) Bishan Park.

4. Streetdirectory (2009) Surin Avenue Neighbourhood Park Fitness Corner.

5. National Parks Board (2013) Recreation & Activities. In: Connector EtltpaKP, editor. Singapore.
